# Operationalization of a multidimensional sex/gender concept for quantitative environmental health research and implementation in the KORA study: Results of the collaborative research project INGER

**DOI:** 10.3389/fpubh.2023.1128918

**Published:** 2023-04-17

**Authors:** Ute Kraus, Katharina Jacke, Lisa Dandolo, Malgorzata Debiak, Sophie Fichter, Katrin Groth, Marike Kolossa-Gehring, Christina Hartig, Sophie Horstmann, Alexandra Schneider, Kerstin Palm, Gabriele Bolte

**Affiliations:** ^1^German Research Center for Environmental Health, Institute of Epidemiology, Helmholtz Zentrum München, Neuherberg, Germany; ^2^Gender and Science Research Unit, Institute of History, Humboldt-University of Berlin, Berlin, Germany; ^3^Department of Social Epidemiology, Institute of Public Health and Nursing Research, University of Bremen, Bremen, Germany; ^4^Section II 1.2 Toxicology, Health-Related Environmental Monitoring, German Environment Agency, Berlin, Germany

**Keywords:** sex/gender, sex, gender, operationalization, intersectionality, environmental health research

## Abstract

**Background:**

In environmental health research, sex and gender are not yet adequately considered. There is a need to improve data collection in population-based environmental health studies by comprehensively surveying sex/gender-related aspects according to gender theoretical concepts. Thus, within the joint project INGER we developed a multidimensional sex/gender concept which we aimed to operationalize and to test the operationalization for feasibility.

**Methods:**

In an iterative process, we created questionnaire modules which quantitatively captured the requirements of the INGER sex/gender concept. We deployed it in the KORA cohort (Cooperative Health Research in the Region of Augsburg, Germany) in 2019 and evaluated response and missing rates.

**Results:**

The individual sex/gender self-concept was surveyed *via* a two-step approach that asked for sex assigned at birth and the current sex/gender identity. Additionally, we used existing tools to query internalized sex/gender roles and externalized sex/gender expressions. Adapted to the KORA population, we asked for discrimination experiences and care and household activities contributing to explain structural sex/gender relations. Further intersectionality-related social categories (e.g., socio-economic position), lifestyle and psychosocial factors were covered through data available in KORA. We could not identify appropriate tools to assess the true biological sex, sexual orientation and ethnic/cultural identity, which have yet to be developed or improved. The response-rate was 71%, the evaluation of 3,743 questionnaires showed a low missing rate. Prevalence of marginalized groups regarding sex/gender identity and definable by experiences of discrimination was very low.

**Conclusion:**

We have shown how the multidimensional INGER sex/gender concept can be operationalized according to an European and North American understanding of sex/gender for use in quantitative research. The questionnaire modules proved feasible in an epidemiologic cohort study. Being a balancing act between theoretical concepts and its quantitative implementation our operationalization paves the way for an adequate consideration of sex/gender in environmental health research.

## 1. Introduction

The differentiation between sex and gender has been around for many decades and the importance of both areas for health is well known (1). Various scientific papers advise that both should be taken into account for better science and health equity (2–7), especially in environmental health research (8, 9). This research field lacks answers to the questions of whether and if so, which dimensions of sex and gender lead to particularly high or low levels of exposure, and whether the exposure-response association is different between sex or gender groups. Additionally, if a commonly used binary variable sex or gender shows up as an effect modifier of an exposure-response association, it has to be clarified which exact dimensions of sex and gender explain this effect modification.

Gender refers to social power relations of segregated gendered groups and includes socially influenced behaviors and living conditions and thus differences in e.g., activity patterns, microenvironments and diets. Therefore, gender can directly influence the environmental exposure on the individual and structural level. On the other hand, the health effects of environmental exposures are related to the biological sex, which is composed of genetic, anatomical, hormonal, and physiological characteristics at a certain time in a social world (5, 10). To underpin the entanglement of social and biological dimensions, the term “sex/gender” is increasingly used (11). However, a dichotomous category “male/female” is usually used for subgroup analyses or just to include it as confounder in multivariable analyses, assuming static differences between men and women on an individual level. This is accompanied by the lack of methods to adequately survey sex and gender (12). New strategies for surveying gender and gender diversity have been increasingly discussed in the political and social sciences (13, 14), especially in the field of survey research (15, 16), as well as in the health sciences (17–20). Thereby, continuous gender-rating scales (13), gender scores (20, 21) or individual gender-related variables (19) are used. Also a diagram was published that can map the complexity of sex/gender identity beyond the binary (22, 23). In addition, the field of quantitative intersectionality takes up possibilities of how the specific structural life circumstances of vulnerable groups can be operationalized and analyzed quantitatively (24, 25). However, there is still a lack of gender-sensitive quantitative methods, which are based on a comprehensive gender-theoretical concept, to analyze the influence of sex and gender on environmental health. A broader scientific debate about how to more appropriately capture biological sex is missing too. Therefore, within the collaborative research project INGER (“Integrating gender into environmental health research: Building a sound evidence basis for gender-sensitive prevention and environmental health protection”) we developed a new sex/gender concept which is described in details elsewhere (26). Briefly, the concept is based on four theory-driven prerequisites derived from biology, medicine, public health and gender studies to adequately describe sex/gender in all its complexity based on current research. These are multidimensionality, variety, intersectionality and embodiment. Thus, the concept moves beyond a binary to a multidimensional view of sex/gender as it defines the individual sex/gender self-concept by various dimensions: the sex assigned at birth, the sex phenotype at birth and at the current time, current sex/gender identity, internalized sex/gender roles and externalized sex/gender expressions. Variety—the variability of sexed and gendered groups—is taken into account as all of these described dimensions of sex/gender can be combined in different ways (e.g., feminine identity, masculine expression, and gender neutral/egalitarian roles). Sex/gender is seen as dynamic, not as a static state and all dimensions influence each other and can change dependent on the context. Corresponding to the embodiment theory, which describes the biological incorporation of the social (27), we assume that somatic and social factors dynamically influence each other and therefore cannot be conceptualized as independent of each other. In the sense of intersectionality as a theoretical framework, the concept assumes that societal sex/gender positions are heterogeneous and determined by interacting social strata, e.g., family situation, education or occupation. Thereby, sex/gender is seen as shaped by societal power relations including discrimination and stress experiences, social support, care, and household activities and health-related behavior.

To address this theoretical concept in quantitative research, we developed questionnaire modules within INGER and assessed their feasibility in two established frameworks for environmental health research: the German Environment Specimen Bank (ESB) (28) operated by the German Environment Agency, and the cohort of the Cooperative Health Research in the Region of Augsburg (KORA) operated by Helmholtz Munich, Germany (29). This article describes the operationalization of INGER's multidimensional sex/gender concept and presents the results of the descriptive analysis of the questionnaire data collected in the KORA cohort. Further, we discuss the resulting benefits of including sex/gender-related dimensions in quantitative research based on cohort studies.

## 2. Development of the questionnaire

### 2.1. Iterative process

In a consensus process, the INGER research consortium designed questionnaire modules that largely meet the requirements of the sex/gender concept which was developed in a parallel process. This process was based on a synopsis of previous sex- and gender-related approaches and their operationalization examples (26) as well as on further extensive research on possible survey tools on sex and gender. Fundamental to the development was a comprehensive decision-making process between the experts of the scientific consortium including the scientific advisory board. For the implementation of survey tools, an iterative discussion procedure was initiated, inspired by the nominal group technique (30). Initially, possible survey tools and their impact and historical context were presented in an internal gender-theoretical discussion paper. A second discussion paper from a gender-theoretical perspective weighed up the advantages and disadvantages of the different instruments. The consortium members also contributed further instruments to the discussion process and voted on all instruments in a consensus process during several discussion rounds in 2018. The scientific advisory board of INGER debated a first version of the questionnaire modules in spring 2018. The piloting took place in autumn 2018.

### 2.2. Sex/gender-related facets to be operationalized

#### 2.2.1. Individual sex/gender self-concept

We see the individual sex/gender self-concept as being shaped by several dimensions (26): sex assigned at birth, sex phenotype at birth and at the current time, current sex/gender identity, internalized sex/gender roles and externalized sex/gender expressions. Sex/gender identity marks how individuals position themselves in terms of masculinity, femininity or beyond. The sex/gender identity influences sex/gender roles and thus feelings and behaviors and sex/gender expression (5). Internalized sex/gender roles are understood as internalized behavioral norms that differ for sex/gender groups and have consequences for a person's social position. They influence the sense of self, expectations and experiences, self-confidence, choice of profession, and social position in a hierarchical social system (5). Externalized sex/gender expressions characterize the visual self-presentation (of a specific sex/gender position), thus influence how a person is perceived and perceives him/herself—conforming or non-conforming to existing sex/gender norms.

#### 2.2.2. Structural sex/gender relations

Sex/gender relations describe how people interact and are treated based on their sex/gender. These sex/gender relations open up life chances or can exclude from participation, they are differentiated according to further intersecting categories of social distinction such as ethnicity, socio-economic position, disability and sexual orientation and are characterized by a hierarchy (intersectionality). The sex/gender self-concept is significantly influenced by these sex/gender relations in that they reveal social power relations and highlight structural living conditions for very different sex/gender groups (e.g., white heterosexual men with high education and high income, white working class women with middle income and low education, lesbian black academics with low income, female Muslim immigrants whose education is not recognized and who have no legal income and residence status or trans people whose transition process might be influenced by their socio-economic position). Sex/gender relations differ according to the social position of the members of the gender groups and vice versa. In order to do justice to the concept of intersectionality, the interconnectedness of different social positions must be specified. This requires the operationalization of a set of parameters to characterize further relations of social inequality which intersect with sex/gender dimensions.

#### 2.2.3. Life-style and psychosocial factors

Life-style and psychosocial factors have a significant influence on health. This includes detrimental aspects such as smoking, alcohol consumption, lack of exercise and stress as well as health resources such as social support or a healthy diet. These health-relevant factors have so far been operationalized predominantly on the individual level, but arise as a consequence of individuals' social circumstances, norms, roles and socialization conditions and might therefore be gender-related (31, 32). This makes life-style and psychosocial factors relevant as parts of the INGER sex/gender concept.

### 2.3. Final questionnaire

The final questionnaire was developed in German, but the individual questions and response categories are translated into English here. The individual sex/gender self-concept was developed for general application in various fields such as epidemiological studies or human biomonitoring research. The same is true for the topics that were considered important for contributing to explain structural sex/gender relations. However, items selected for each topic may vary depending on the target population.

#### 2.3.1. The individual sex/gender self-concept

We assessed sex/gender in a two-step approach. We asked for the sex assigned at birth with the in Germany legal possible entries “female”, “male”, and “diverse/intersexual”. Current sex/gender identity could be categorized as “female”, “male”, “trans^*^/transman/transwoman”, “Inter^*^”, “an identity not mentioned here” or “I do not want to classify as any sex/gender category”. Categories were based on the report of the Federal Anti-Discrimination Agency in Germany (FADA) (33) and the two-question method for assessing gender categories validated for the US by Tate et al. (34). We measured internalized sex/gender roles by using the first two items of the Traditional Masculinity and Femininity (TMF) Scale (35), which asked (1) how feminine or masculine the participants perceive themselves to be and (2) how they would ideally like to be. Answers could be indicated on a 7-point scale from “very masculine” to “very feminine”. Kachel et al. (35) define femininity and masculinity as “characteristics encompassing traits, appearances, interests, and behaviors that have traditionally been considered relatively more typical of women and men, respectively.” We agree with this vague definition. However, terms were not explained to the participants, as the instrument was intended to measure the extent to which the respondents are in conflict with gender-specific self-attributions and attributions by others. The participants therefore gave answers based on their own understanding of masculinity and femininity. In addition, participants were asked about their agreement to nine statements about gender roles drawn from the Family and Changing Gender Roles Module of the International Social Survey Programme (36, 37). Externalized sex/gender expressions were queried using two items based on a brief measure describing socially assigned gender (non)conformity (18, 38, 39). Participants were asked how other people would describe them in general, firstly on the basis of their appearance, style of dress and other visual characteristics, and secondly on the basis of their behavior. Again, the answers were given on a 7-point scale ranging from “very masculine” to “very feminine”. For an overview of the operationalization of the multidimensionality of sex/gender see Table 1.

**Table 1:** Operationalization of the individual sex/gender self-concept.

	**Questionnaire item**	**Categories/coding**	**Source**
Sex assigned at birth	“What sex were you assigned at birth?”	• Female • Male • Intersexual	Legal registration options in Germany
Current sex/gender identity	“What is your current sex/gender identity?”	• Female • Male • Trans^*^/transman/transwoman • Inter^*^ • An identity not mentioned here • I do not want to classify as any sex/gender category	Adapted from Beigang et al. (33); Tate et al. (34)
Internalized sex/gender roles	“The following statements relate to how feminine or masculine you perceive yourself to be. Please describe yourself by selecting the answer that best fits you. a) I mostly perceive myself as.. b) Ideally I would like to be…”	7-point scale: very masculine, mainly masculine, little masculine, as feminine as masculine, a little feminine, mainly feminine, very feminine	Kachel et al. (35), first & second item of the TMF scale
Internalized sex/gender roles	9 Statements on attitudes toward gender roles:	5-point scale: strongly agree, agree, neither agree nor disagree, disagree, strongly disagree	
	1. “Both the men and women should contribute to the household income.”		ISSP (36), R2.a; Scholz and Jutz (37), J002.a
	2. “The man's job is to earn money; a woman's job is to look after the home and family.”		ISSP (36), R2.b; Scholz and Jutz (37), J002.b
	3. “A working mother can establish just as warm and secure a relationship with her children as a mother who does not work.”		ISSP (36), R1.a; Scholz and Jutz (37), J001.a
	4. “A pre-school child is likely to suffer if his or her mother works.”		ISSP (36), R1.b; Scholz and Jutz (37), J001.b
	5. “All in all, family life suffers when the woman is working.”		ISSP (36), R1.c; Scholz and Jutz (37), J001.c
	6. “Being a housewife is just as fulfilling as working for pay.”		ISSP (36), R1.e; Scholz and Jutz (37), J001.e
	7. “Being a househusband is just as fulfilling as working for pay.”		Own considerations
	8. “One parent can bring up a child as well as two parents together.”		ISSP (36), N5.a; Scholz and Jutz (37), J005.a
	9. “A same sex couple can bring up a child as well as a male-female couple.”		Modified from ISSP (36), N5.b and c; Scholz and Jutz (37), J005.b and c
Fluidity	“Has your assessment of what is feminine or masculine changed in recent years?”	Yes/No	Own considerations
	“Has your gender identity changed in recent years?”	Yes/No	Own considerations
Externalized sex/gender expressions	“How would other people generally describe you…	7-point scale: very masculine, mainly masculine, little masculine, as feminine as masculine, a little feminine, mainly feminine, very feminine	Modified from Wylie et al. (39)
	… based on your appearance, clothing style, and other visual characteristics?”		
	… based on your behaviors?”		

To assess the changeability and fluidity of the individual sex/gender self-concept, we asked, if (1) participants' assessment of what is feminine or masculine and (2) if their sex/gender identity had changed in recent years (yes/no).

#### 2.3.2. Items contributing to explain structural sex/gender relations

As items contributing to explain structural sex/gender relations with reference to social differentiations within gender groups, we focused on experiences of discrimination as dimensions of social inequalities that can be described as intersected with sex/gender (e.g., discrimination/othering based on ethnicity). Additionally, we assessed frequently sex/gender-segregated care and household activities (Table 2).

**Table 2:** Items contributing to explain structural sex/gender relations that (1) are intersectionally structured by further factors of social inequality: discrimination experiences and (2) represent gendered social positioning: care and household activities.

	**Questionnaire item**	**Categories/coding**	**Source**
Discrimination experiences	“Which statements apply to you? I have the feeling…”	5-point scale: fully applies, rather applies, partly/partly, rather does not apply, does not apply at all	Topics chosen on the basis of Beigang et al. (33); modified from EWCS (40)
	“… that life offers me many opportunities.”		
	“… to be accepted as I am.”		
	“… being heavily involved due to family responsibilities.”		
	“… to be disadvantaged by my position in society”		
	“… to be disadvantaged because of my age.”		
	“… to be disadvantaged because of my height.”		
	“… to be disadvantaged because of my weight.”		
	“… to be disadvantaged because of my physical impairment.”		
	“… to be disadvantaged because of my ethnic/cultural affiliation.”		
	“… to be disadvantaged because of my sex/gender.”		
	“… to be disadvantaged because of my sexual orientation.”		
Discrimination experiences	“Have you ever been asked in Germany whether you or your parents were born abroad?”	Yes/No	Developed based on SVR (41)
	“If so, what characteristics do you think they asked you about?”	• Physical appearance • Clothes • Religious signs • Name • Language accent • Nutrition • other, namely:	Developed based on SVR (41)
Care and household activities	“Who is currently taking primary responsibility for the following tasks? (“Other people” also includes your partner)”	5-point scale: Only me, mainly me, I together with other persons, mainly other persons, only other persons and does not apply	Developed on the basis of ISSP (36); SHARE (n.d.), SOEP (42); EQLS (43) (if not otherwise specified)
	1. “Care and/or upbringing of your children/grandchildren, driving services for your children/grandchildren”		
	2. “Care for disabled, chronically ill or in need of care family members, neighbors or friends”		
	3. “Earning a living (including pension)”		
	4. “Cooking”		
	5. “Housework”		
	6. “Errands (shopping, procurement)”		
	7. “Administrative tasks (insurance, tax return, etc.)”		
	8. Technical activities (e.g., computer, internet)		Own considerations
	9. “Handicraft tasks in the household”		
	10. Gardening (during the gardening season)		Own considerations
Care and household activities	“How often do you engage in these activities?”	5-point scale: daily, several times a week, 1 to 2 times a week, less often, never, not applicable	Developed on the basis of ISSP (36); SHARE (n.d.), SOEP (42); EQLS (43)
	*Same 10 tasks as above*		

EQLS, European Quality of Life Survey; ISSP, International Social Survey Programme; SHARE, Survey of Health, Aging and Retirement in Europe; SOEP, German socioeconomic panel study.

Experiences of discrimination were queried using eleven statements to which participants were asked to indicate their agreement on a 5-point scale from “fully applies” to “do not apply at all”. On the basis of the report of the Federal Anti-Discrimination Agency in Germany (33) and the European Working Conditions Surveys (40) we came up with following aspects which we considered relevant for the KORA population: opportunities in life, self-acceptance, involvement due to family responsibilities, disadvantages because of the position in society, age, height, weight, physical impairment, ethnic/cultural affiliation, sex/gender, and sexual orientation. If participants experienced discrimination due to further reasons they could indicate these as free text. In addition, participants answered the question of whether they had ever been asked in Germany if they or their parents were born abroad and, if so, on the basis of which characteristics (41). Care and household activities were queried in two steps. First we asked who is responsible for different tasks (Table 2) which could be answered with “only me”, “mainly me”, “I together with other persons”, “mainly other persons”, “only other persons” and “does not apply”. In the second step we asked how often the participant engages in these tasks (daily, several times a week, 1 to 2 times a week, less often, never, not applicable). The selection of these tasks was based on various national and international surveys: ISSP (36), Survey of Health, Aging and Retirement in Europe (44), the German socioeconomic panel study (42) and the European Quality of Life Survey (43) (see also Table 2).

In order to address further intersectionality-related social categories we could refer to available variables of the KORA study which included family situation, education, occupation, employment, income, socio-economic status, ethnicity and disability. For detailed information on variables and categories see Supplementary material S1. Regarding lifestyle and psychological factors we could use already existing data on smoking status, alcohol consumption, physical activity, stress and self-efficacy (Supplementary material S2).

## 3. Implementation of the questionnaire in the KORA cohort

### 3.1. Methods

#### 3.1.1. Study population and field phase

KORA is a research platform to examine the prevalence and incidence of various chronic diseases in the general population of Augsburg, Germany, and two adjacent counties (29). Since 1984, four cross-sectional surveys at 5-year intervals and several follow-up studies were conducted within KORA and comprised 18,079 participants. The four surveys took place in 1984/85 (S1, participants born between 1920 and 1959), in 1989/90 (S2, 1915–1964), in 1994/95 (S3, 1920–1969) and in 1999/2000 (S4, 1925–1975). The INGER questions were part of a 24-page questionnaire that also asked about participants's health, the living environment including green and blue spaces, the home including housing quality and perceived environmental noise, noise reduction activities and employment as part of another project. The paper-based questionnaire was sent to all participants of the follow-up study “KORA FIT”, which was conducted in 2018/2019 and for which the participants of all four KORA surveys born between 1945–1964 were eligible. Additionally, all younger participants of S3 (born between 1965 and 1969) and all younger (born between 1965 and 1975) and older (born between 1925 and 1944) participants of S4, who did not participate in KORA FIT, were invited to fill in the INGER questionnaire. For an overview of eligible participants see Supplementary material S3. The INGER questionnaire was sent out in parallel with the KORA FIT study in two waves, the first in February 2019 and the second in July 2019. In order to achieve a high response rate, a reminder was sent by postcard after 2 weeks to all those who had not yet returned the questionnaire.

The study methods were approved by the Ethics Committees of the Bavarian Chamber of Physicians (KORA-Fit EC No 17040). All study participants gave their written informed consent.

#### 3.1.2. Statistical analyses

We calculated means and standard deviation or absolute and relative frequency counts, respectively, of baseline characteristics of the study population and variables related to the INGER sex/gender concept and calculated its correlations. Building on previous calculations (18, 38, 39, 45) we assessed socially assigned gender (non)conformity by calculating different measures. In a first step, scale values regarding internalized sex/gender roles and externalized sex/gender expression with each two variables were recoded based on participants' sex assigned at birth. For male participants, we coded “very masculine” (coding 1) as “very conforming” (1) up to “very feminine” (7) as “very non-conforming” (7). For females, “very masculine” (1) was coded as “very non-conforming” (7) up to “very feminine” (7) coded as “very conforming” (1). Thus, socially assigned gender (non)conformity measures ranged from 1 to 7, with low values indicating high gender conformity and high values indicating high gender non-conformity. In a second step, in line with Hart et al. (18) we formed (non-)conformity as a dichotomous variable, with “conforming” corresponding to scores below or equal to 4 and “non-conforming” to scores above 4. We did this on the one hand for the mean of both variables regarding the external sex/gender expression and on the other hand for the self-rated internal sex/gender role. Statistical analysis were performed with R (version 4.1.2).

#### 3.1.3. Feasibility

We used the response rate and proportions of missing values of single questions as indicators of feasibility of the operationalization of the multidimensional sex/gender concept.

### 3.2. Results

#### 3.2.1. Overall response rate and acceptance

The questionnaire was sent to 5,256 eligible KORA participants. Of these, 25 were unknown moved and 13 had moved outside the study area, 83 were deceased, 36 had to be excluded due to health restrictions and 1,321 refused to participate and either did not return the questionnaire at all or without a signed data use agreement. One participant withdrew consent. Finally, 3,742 questionnaires were available for analysis, corresponding to a response rate of 71.2%. Due to the limited number of pages in the questionnaire, we had not included an extra field to ask participants to comment on the survey. Nevertheless, few participants (*N* = 49, 1.3%) gave unsolicited comments next to individual sex/gender questions. The comments included questions about the definition of masculine and feminine, and what is meant by the corresponding questions. Others titled the questions as “nonsense.” On the other hand, some participants gave positive comments on the sex/gender issue (Supplementary material S4).

#### 3.2.2. Results of descriptive analyses

Mean age of the study participants was 63.8 years (standard deviation: 9.4) with a range of 43 to 93 years. The frequency distribution of sex assigned at birth and the current sex/gender identity at time of survey is shown in Table 3. Regarding their sex assigned at birth, 2,014 participants were female, 1,684 were male. No participant indicated to have an intersexual sex assigned at birth, which corresponds to the fact that this specification was not yet legally possible in Germany before 2018. The current sex/gender identity was largely consistent with the sex assigned at birth (Cramer's V coefficient for correlation: 0.75). However, two participants stated their current sex/gender identity as trans^*^/transman/transwoman. Four further participants could be described as transgender as well, but these reported their current sex/gender identity as male with female sex assigned at birth (*N* = 2) or as female with male sex assigned at birth (*N* = 2). Four participants had an identity not mentioned and 14 did not want to classify themselves as any sex/gender category. In terms of sex/gender fluidity, 44 participants (1.2%) indicated that their sex/gender identity had changed in recent years.

**Table 3:** Frequency distribution of sex assigned at birth and the current sex/gender identity.

**Current sex/gender identity^a^**	**Total**		**Sex assigned at birth**			
			**Female**	**Male**	**Intersexual**	**Missing**
**Total**	**3,742**	**(100%)**	**2,014 (53.8%)**	**1,684 (45%)**	**0**	**44 (1.2%)**
Female	**1,998**	(53.4%)	1,981	2	0	15
Male	**1,650**	(44.1%)	2	1,640	0	8
Trans^*^/transman/ transwoman	**2**	(0.1%)	2	0	0	0
Inter^*^	**0**	(0%)	0	0	0	0
An identity not mentioned here	**4**	(0.1%)	0	4	0	0
I do not want to classify as any sex/gender category	**14**	(0.4%)	2	11	0	1
Multiple entries	**14**	(0.4%)	3	11	0	0
Missing	**60**	(1.6%)	24	16	0	20

^a^At time of survey. Bold values are total number counts (N).

With regard to the internalized sex/gender role, the most frequently indicated categories, each with more than 20%, were “mostly masculine” and “mostly feminine”. In total 10.8 and 11.8% of participants stated to perceive themselves as “very masculine” or “very feminine”, respectively. When asked how they would ideally like to be, these percentages were higher (12.9 and 14.9%). Slightly more than 10% answered both questions with “as feminine as masculine” (11.4 and 10.3%). In terms of their externalized sex/gender expression, participants most frequently indicated that other people would describe them as “very masculine” (23.6%) and “very feminine” (27.0%) based on their appearance, dress style, and other visual characteristics. These percentages were similar and slightly lower, respectively, for the question of how others would describe them based on their behavior (23.4 and 25.5%). The assessment of what other people would think was more often stated as just “as feminine as masculine” regarding the behavior than regarding the appearance, style of dress and other visual characteristics (12.2 vs. 8.5%; Supplementary material S5). The question of whether the assessment of what is feminine or masculine has changed was affirmed by 382 participants (10.2%).

**Figure 1:**
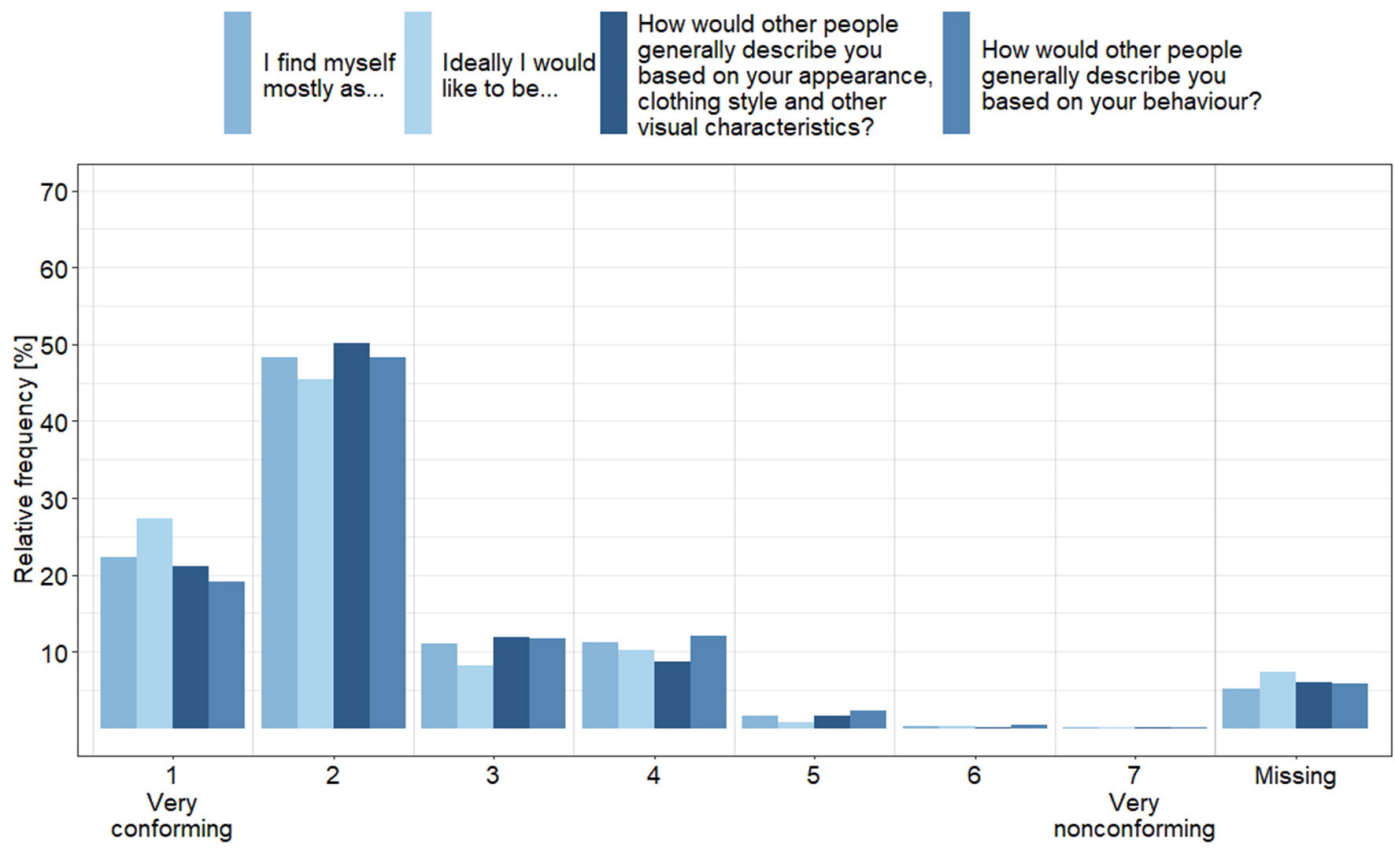
Socially assigned gender (non)conformity, based on sex assigned at birth, separately for the two items for internalized sex/gender roles and the two items for externalized sex/gender expression, which were answered on a 7-point scale from “very masculine” to “very feminine” (*N* = 3.742).

Figure 1 shows the socially assigned gender (non)conformity, a measure of the degree to which participants' internalized sex/gender roles and externalized sex/gender expression match their sex assigned at birth. Less than one third of participants showed absolute gender conformity (score 1) related to the corresponding four items ranging from 19 to 27%. Around half of participants had a high gender conformity (45–50%, score 2), but 18–24% of participants could be classified as moderately non-conform (score 3 and 4) and 1–3% as very non-conform (scores > 4). With regard to the dichotomous variable conforming vs. non-conforming, we observed 93 (2.5%) participants who assumed that others would view them as non-conforming. Of these, 39 persons (42%) classified themselves also as non-conforming, while 54 saw themselves as conforming (Supplementary material S6).

**Figure 2:**
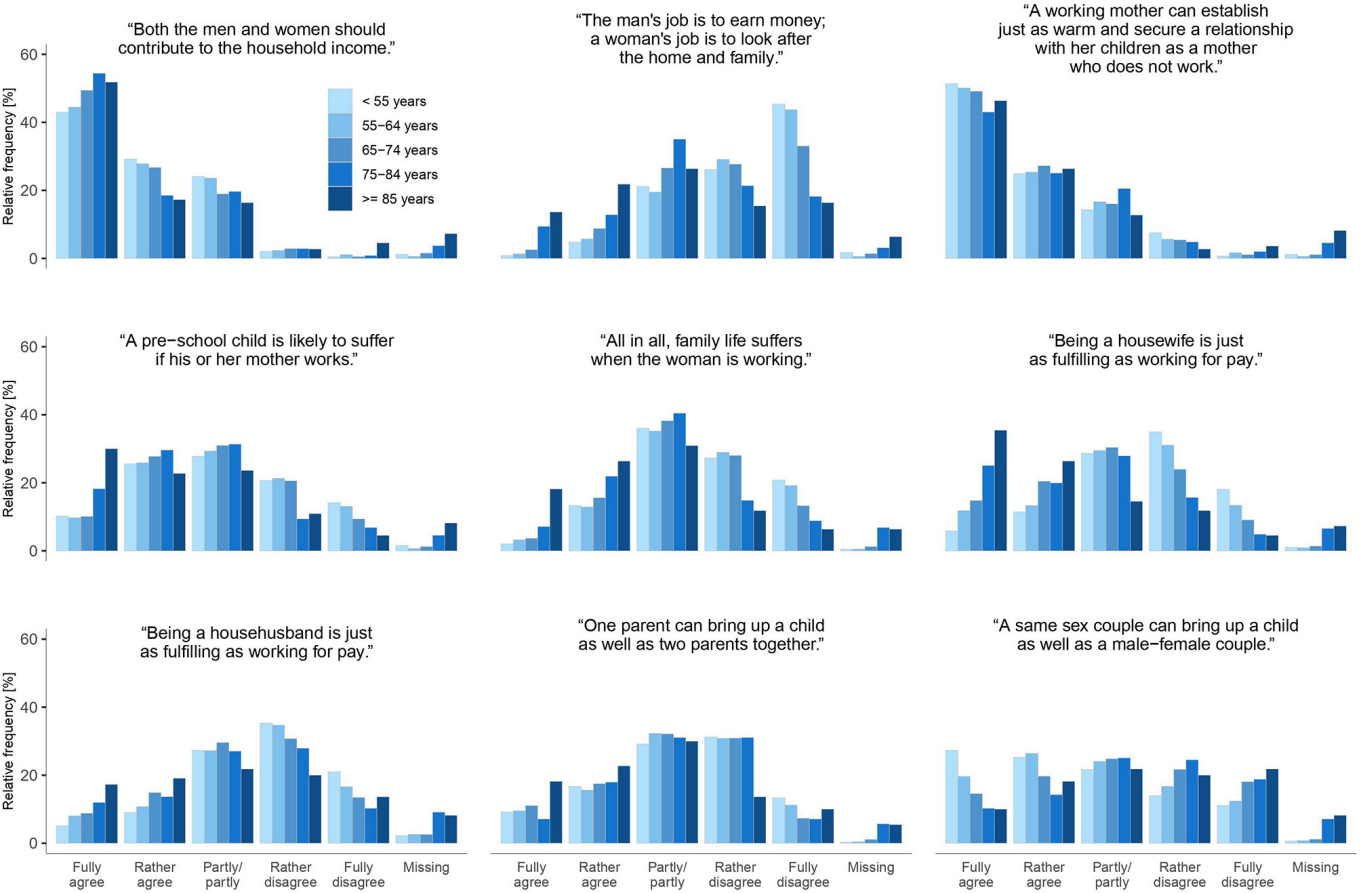
Attitudes toward gender roles by age-group (*N* = 3.742).

Figure 2 displays the frequencies of agreement with gender role attitudes separately by age group. Results showed that participants generally held egalitarian rather than traditional attitudes, with this being more pronounced in the younger age groups. For example, the majority of participants rather or fully disagreed with the statement “The man's job is to earn money; a woman's job is to look after the home and family” (*N* = 2,390, 64%, Supplementary material S7). Of these participants, however, most were of younger age, while the majority of those who fully or rather agreed were of older age. The study participants felt rather not or not at all discriminated with regard to most aspects (Supplementary material S8). Most participants did not feel discriminated against because of sexual orientation (does not apply at all: 90%). Social position and age were the least rejected reasons for discrimination (does not apply at all: 45.9 and 43.6%, respectively). The question, if participants had the feeling to be disadvantaged by other reasons, was answered in the affirmative by 104 persons. As topics not already queried by us, the family situation (e.g., not being married, not having children, and being widowed), political views, mental illness, and personal characteristics were mentioned as reasons for discrimination as free text.

**Figure 3:**
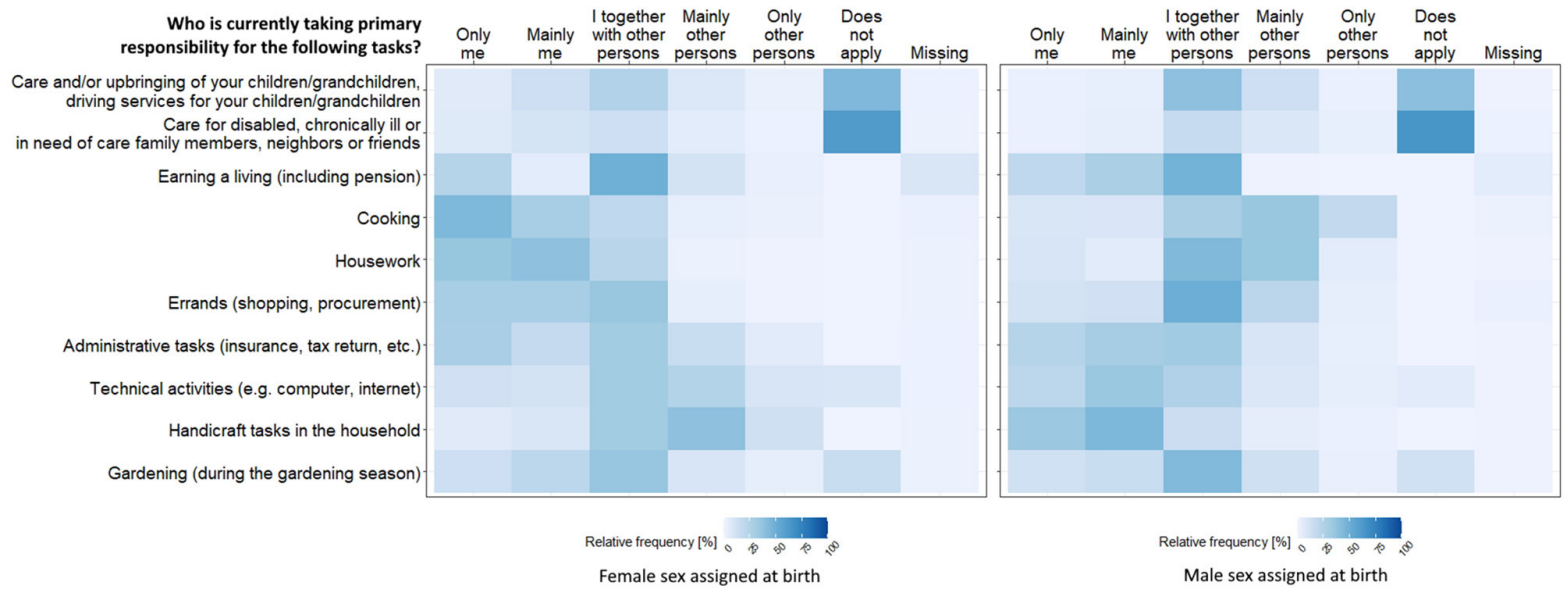
Relative frequencies of responsibilities for care and household activities, separately for female and male sex assigned at birth (*N* = 3.742).

Figure 3 shows the relative frequencies of care and household activity tasks separately for male and female sex assigned at birth (for the whole sample see Supplementary material S9). A traditional division of roles can be identified. Areas such as childcare, cooking, housework and shopping are more often seen as the sole responsibility of participant with female than with male sex assigned at birth. For example cooking was answered with “only or mainly me” by 73.8% of persons with female vs. 17.6% of persons with male sex assigned at birth. In contrast, areas such as administrative tasks, technical and manual activities were more often the responsibility of participants with male sex assigned at birth (e.g., handicrafts in the household: 78.5% of males vs. 15.0% of females). For the frequency distribution of intersectionality-related social categories and lifestyle and psychosocial factors see Supplementary material S1, S2. Because these items were taken from existing data collected primarily in the KORA FIT study, they were available only for a subset of INGER participants.

#### 3.2.3. Question specific missing rates

We evaluated the feasibility regarding our questions on sex/gender-related items *via* the missing rates (Supplementary material S10), which were generally low. There were 1.2% missing data for sex assigned at birth and 1.6% missing data for current sex/gender identity. Highest missing rates occurred with respect to the questions asking participants to indicate how they rate themselves on a continuum from very masculine to very feminine (range: 4.7–6.9%). Accordingly, the question whether the assessment of what is male and female has changed in recent years was not answered by 4.1% of the participants. Missing rates for variables regarding gender role attitudes ranged from 1.3 to 1.8%, except for the item “Being a househusband is just as fulfilling as working for pay.” which stood out at 3.3%. Missing rates ranged from 0.9 to 1.4% for all items of discrimination, except for discrimination due to sexual orientation with a missing rate of 2.2%. Missing rates of care and household activity variables ranged with one exception from 1.1 to 1.8%. Only the question about who is responsible for earning a living had a high missing rate of 7.3%. This mainly affected pensioners (78%), who probably did not understand that drawing a pension is to be considered a wage.

For most of the variables already available in KORA (Supplementary material S1, S2) the missing rates were comparable. However, for questions asked in a face-to-face interview they were even lower in some cases. For data collected in a self-completion questionnaire, missing rates were similar or even higher (e.g., net household income: 7.4%).

## 4. Discussion

Within the interdisciplinary research project INGER, the newly developed multidimensional sex/gender concept based on current gender theory (26) was for the first time operationalized for quantitative environmental health research. The feasibility was successfully tested in the KORA cohort, an epidemiologic study with comprehensive assessment of environmental exposures. In the following, we will discuss advantages and limitations of the operationalization from an interdisciplinary perspective including social science and gender studies. In addition, recommendations for future research are derived from the experiences of the implementation in KORA.

### 4.1. Operationalization of the individual sex/gender self-concept

The inner circle of the graphic of INGER's multidimensional sex/gender concept, the individual sex/gender self-concept, has five dimensions: sex phenotype at birth/sex assigned at birth, current sex phenotype, sex/gender identity, internalized sex/gender roles, externalized sex/gender expression (26).

In our society, sex is assigned at birth based on the observable phenotype and influences all other dimensions without determining them. We assessed sex/gender as a two-step approach enabling the investigation of external attribution (sex assigned at birth) and self-categorization (current sex/gender identity). This approach assesses more than two forms of sex/gender (e.g., female, male, inter^*^, trans^*^) and that a sex/gendered body at birth does not necessarily match a corresponding sex/gendered identity at the time of the study in adulthood. The category “sex assigned at birth” enables comparability with other studies that measure sex/gender with a binary category (male/female) by including the legal sex/gender status. In addition, self-categorization allows space for individual sex/gender identity conceptions. The two-step approach has been evaluated repeatedly in recent years (17, 34, 46) and is now increasingly recommended and used (33, 47–49), allowing for a more accurate mapping of sex/gender minority positions. It was pointed out, that despite slightly different categories, all two-step approaches capture three dimensions of sex/gender: sex assigned at birth, sex/gender identity and trans status (17). Response options used in INGER included “I do not want to classify as any sex/gender category”, which considers individuals who do not identify as any of the other given response categories. However, to overcome the binary construct to more appropriately reflect sex/gender diversity an explicit classification “non-binary” should be added in future studies. An interesting new instrument to map and visualize diverse sex/gender identities is a diagram published by Beischel et al. (23). In addition to cis and trans, it also classifies a category of allogender (neither cis nor trans) and distinguishes between binary and non-binary identities of cis, trans and allogender persons. However, a 12-min instructional video must be watched before use, during which 50% of participants dropped out in the corresponding feasibility study (23). In addition, 19% of the feedback on the instrument from the fully participating participants was negative (22). Therefore, this instrument seems to be inappropriate for the use in quantitative epidemiological studies. For our questionnaire, it would be useful to additionally explain the terms cis, trans^*^, inter^*^, and non-binary to all study participants.

The use of the two-step approach in data collection proves feasible in the KORA cohort. Missing rate was extraordinary low and comparable to other sex/gender- and not directly sex/gender-related variables in our survey. As expected, prevalence of non-cisgender persons, i.e., persons with a current sex/gender identity not matching their sex assigned at birth, was very low in our study (0.45%). This result based on the KORA cohort should not lead to a general conclusion that the two-step approach is superfluous in epidemiological studies. The observed prevalence seems to reflect the reality of the distribution of non-cisgender persons, as it is comparable to or even higher as the prevalence estimates of other only rare, population-based surveys, that were predominantly conducted in the United States (50–52).

To measure internalized sex/gender roles, we used the TMF scale, an instrument from psychology—new at the time of questionnaire development—which considers femininity and masculinity as higher order constructs (35). Sex/gender roles are usually measured in psychological studies by scales for personality attributes, behaviors, interests, and attitudes with the Bem sex role inventory as one prominent example (12, 53). The TMF scale allows self-categorization without assigning stereotyping and culture-specific concrete traits and is thus also internationally valid in different cultural areas. In addition, it easily separates the constructs of masculinity and femininity. As sex/gender roles are constantly changing, trait-based scales can no longer reliably assign traits to different sex/genders (35, 54). While this instrument leaves open which concrete traits a respondent associates with femininity and masculinity, it may reveal an internalized sense of femininity and masculinity as a characteristic of sex/gender. However, the TMF scale assumes binarity which we basically wanted to overcome. Nevertheless, it helps to investigate whether measurable sex/gender health differences can be related to the self-concept rather than the sex assigned at birth (e.g., risk taking behavior—which is assumed to be a masculine trait—in females with a low feminine sex/gender role concept (55).

To further assess a person's gender role attitude, we applied items of the proven instrument of the ISSP, suitable in content for the middle-aged to older persons of the KORA population (36, 37). However, some items of the instrument are not entirely clear in their construct validity: e.g., “One parent can bring up a child as well as two parents together” and “A same sex couple can bring up a child as well as a male-female couple.” Both statements could also be rejected when structural barriers impair equal opportunities. Nevertheless, this instrument allows us to look at the influence of internalized gender roles on health. In particular, the discrepancies between sex/gender role attitudes and actual gender role behavior could have a negative effect on the health-related quality of life if these discrepancies lead to subliminal stress and in a long run to an increased allostatic load. Cumulative allostatic load has been discussed as one major biological pathway linking social inequalities with ill health (56, 57). However, effects of gender role attitudes and behavior on health status have not yet been the focus of health research and should therefore be investigated more intensively in the future.

In general, the participants in our survey tended to have a more egalitarian attitude. But we observed greater proportions of traditional attitudes in the older age groups, which suggests that gender role attitudes adjust over time with societal changes. With respect to the feasibility, missing rates was low except for the question if being a househusband is just as fulfilling as working for pay. Of the participants who did not answer this question, 74% had a female sex/gender identity who may have found it difficult to answer this question from a rather unusual househusband's perspective.

Externalized sex/gender expression as perceived by others was assessed based on a short self-report measure for use in the study of health disparities (39). Combined with sex assigned at birth it can be used to evaluate sex/gender non-conformity (18, 38, 39, 45) which may be a source of detrimental health effects. Individuals who are perceived by society as sex/gender non-conforming and thus deviate from an expected appearance may suffer from long-lasting psychological distress and somatic complaints because of discrimination (58, 59). Accordingly, non-conforming adolescents have been shown to have a lower health-related quality of life (38) or lower self-reported health (18). In our analysis, we evaluated the non-conformity based on the external attribution as well as on the internalized sex/gender roles. In line with Hart et al. (18), we found in the KORA population that less than one-third of participants selected the highest category (very masculine or very feminine) for each item that corresponded to their sex assigned at birth. Additionally, Hart et al. (18) found that only people for whom the self-rated non-conformity did not match the assumed appraisal by others reported poorer self-related health. When we classified our participants either as conforming or as non-conforming, 1% assumed to be viewed as non-conforming by others but had an own perception of conformity and therefore may experience distress and on the long-run health impairments. This underscores the benefit of moving beyond a binary categorization, that does not capture the different degrees within femininity and masculinity. It should be noted that the corresponding items had slightly higher missing rates (4.7–6.9%) in our survey. Although unsolicited comments are not representative for the entire study population, they indicated that the terms “masculine” and “feminine” were sometimes questioned or not understood. When using these scales in the future, the terms should be explained. Another reason could also be the use of a bipolar scale from “very masculine” to “very feminine”. The assessment of the extent of masculinity and femininity may vary in different situations and may not necessarily be mutually exclusive (39). Therefore, we suggest changing the response scales in upcoming studies. Instead of a bipolar scale, two separate scales should be used, as it was also applied recently by Hart et al. (18). One scale should range from “not at all feminine” to “very feminine” and one from “not at all masculine” to “very masculine”. Thus, gender as a bipolar construct is replaced by a more contemporary design that allows for femininity and masculinity at the same time. It also better accounts for non-binary individuals who do not identify as either feminine or masculine, who might answer “not at all” to both scales.

### 4.2. Operationalization of sex/gender relations

In order to adequately map hierarchized social relations between different sex/gender groups, social context factors can add information on structural living conditions leading to social inequalities between diverse population groups. For health, the perceived burden of disadvantage is particularly important (60). Therefore, we used an instrument to measure experiences of discrimination due to several reasons. In general, the study participants felt rather not or not at all discriminated against. As is known from anti-discrimination research in the social sciences, experiences of discrimination, however, do not adequately reflect the actual reality of inequalities. Often, affected persons may not directly recognize disadvantages and individual feelings of disadvantage can be less pronounced than the disadvantage of one's own social group (61, 62). This is in line with the low level of feelings of disadvantage because of sex/gender in the comparatively affluent KORA research population, despite the evidence that more generally at the societal level women are discriminated against according to objective measures such as the Gender Pay Gap.

The INGER assessment of discrimination allows for the consideration of subjective experiences that may have a direct effect on health or may alter vulnerability to environmental exposures. In order to assess the whole spectrum of discrimination and to overcome the data gap regarding marginalized groups in health science, their representation in processes within the health care system (e.g., diagnosis and treatment frequency) should also be examined. However, for the German-speaking area, a consensus on appropriate instruments for surveying identities of marginalized groups (e.g., certain sex/gender identities, cultural/ethnic identity, and sexual orientation) is still missing, but would certainly be necessary. Measuring external attribution is particularly useful for surveying discrimination, which is why we included the question “Have you ever been asked in Germany whether you or your parents were born abroad?”, an instrument on self-assessed othering processes (41). It shows whether a respondent is segregated from the collective of the community and allows for asking the presumed individual characteristics as cause for the question. Such measurement tools are still missing for other reasons of discrimination. Respondents in our study reported little experience with othering processes, which may be because only persons with German citizenship were invited to take part in the four KORA surveys between 1984 and 2000.

One of the greatest inequalities in sex/gender relations at least in the society of Germany is the gender care gap [Second Equality Report of the Federal Government, (63)]. Correspondingly, in our study we observed that women more often had the sole responsibility for childcare or care of disabled people in the personal environment, housework, cooking and shopping. The gender care gap can lead to unequal physiological and psychological distress (64, 65). In addition, this distribution of care and household work has an unequal impact on people with different socio-economic positions. For example, household and care work in high social classes is mainly delegated as paid work to (immigrant) women from low social classes and only people from higher economic positions can afford to delegate household and care work (66, 67).

An adequate analytical integration of sex/gender relations can only take place if structural living conditions such as discrimination, care and household work are included in health studies. The INGER study has operationalized essential aspects here for the KORA population, but further items should be included or developed that capture sex/gender relations more in depth and allow for further differentiation between marginalized groups depending on the specific population group being studied (e.g., job roles, access to public/private childcare, residence status, access to medical care, geodata on crime rates/social housing in the respondents' neighborhood, perceived safety, body sovereignty).

### 4.3. Strengths and limitations

A major strength of our comprehensive sex/gender questionnaire is that we operationalized all items according to a theory-based sex/gender concept that reflects the current state of knowledge on integrating sex/gender in health research. In addition, the response rate was very high, emphasizing the feasibility at least in middle-aged and older population-based cohorts. Furthermore, the operationalization of the sex/gender concept can be applied particularly well to already existing populations. It is possible to draw on already existing data fitting to the concept (especially intersectionality-related social categories), which has a positive effect on the required length of the questionnaire. In particular, the operationalization of the concept was implemented in such a way that all groups, including marginalized ones, are adequately taken into account in data collection. However, existing cohorts might not reflect the diversity of the general population in terms of immigrant groups, low-income groups or people with marginalized gender and sexuality in sufficient case numbers to be able to perform quantitative analyses e.g., on their structural sex/gender relations. This was also the case for our study population with very low or even no persons belonging to marginalized sex/gender identity groups. Therefore, when planning a new study with new recruitment of study participants and depending on the specific research question, an appropriate group-specific power calculation should be made and strategies for contacting and motivating all relevant population groups should be determined (68). Lastly, we collected data on characteristics related to social inequalities separately for relevant categories (e.g., separate questions on sex/gender, socio-economic position) to allow for the application of a variety of statistical methods to analyze the complex interactions of social categories.

Nevertheless, there are also a few limitations of our study. We were not able to include a measurement for the biological sex in our study. The question on sex assigned at birth, which aims to describe an unchangeable state of sex, corresponds to a cultural attribution practice in which an external appearance of a body is categorized according to social standards. Thus, it does not describe all of the physiological details and variability within sex groups and is therefore not meaningful enough as the true biological sex. For a more differentiated assessment of sex, biological measurements (e.g.„ of chromosomes, hormone concentrations, metabolic processes) would be necessary depending on the research question (12), which could not be implemented within the framework of our data collection by self-administered questionnaires.

The questionnaire instruments originate from a European and North American context. Therefore, our operationalization might not be applicable for e.g., indigenous concepts of sex/gender (69) as well as sex/gender relations from South America, Asia or Africa.

To address the presumably low prevalence of marginalized groups, we reemphasise the importance of planning sufficient case numbers when establishing a new study population. This will also show whether the operationalization leads to the same results in terms of response and missing rates as in our middle-aged to older cohort. Furthermore, on the basis of our research interest in health effects of environmental exposures, we collected data on perceived discrimination due to sexual orientation or ethnic/cultural identity as possible causes of chronic stress, but did not also include items on self-assessment of sexual orientation and of ethnic/cultural identity in our questionnaire. Analyses stratified further by categories of sexual orientation or ethnic/cultural identity may be relevant in sex/gender related health (70). However, data collection on sexual orientation has been rather uncommon up to now in German epidemiological studies (71) and community-based developed instruments for both areas are currently still lacking in Germany. In the context of research on discrimination processes, first suggestions to measure sexual orientation (72) and cultural/ethnic identities (47, 61) in German surveys have been made. Involving community members as active and equal participants in the questionnaire development process would ensure that relevant categories are captured for all, including marginalized groups (73). Such a community-based participatory approach would help to develop more comprehensive instruments for data collection, but this was clearly outside the scope of our research aim in INGER.

### 4.4. Outlook: Implications for quantitative statistical analyses

Intersectionality focuses on the close entanglement of social categories and thus interactions of social inequalities (74, 75). The often explorative—analysis of complex interactions of a wide range of social dimensions calls for new statistical approaches (24, 76). In environmental health research in particular, there is a need for methods that can be used to analyze in an intersectionality-informed way whether sex/gender, captured by multiple sex/gender-related variables, has an impact on an environmental exposure or on the association between environmental exposure and a health outcome (8, 77). By combining manifold newly collected data based on the multidimensional INGER sex/gender concept with already available data for an intersectional perspective, the operationalization for the KORA population provides an innovative data base for such analyses. For example, within the INGER project we were recently able to show that decision tree analyses are useful for exploring the relevance of multiple sex/gender dimensions, further social categories and their interactions for the exposure to green spaces (78).

## 5. Conclusion

We demonstrated how the theory-based multidimensional INGER sex/gender concept can be operationalized for quantitative research embedded in European and North American culture and successfully tested the operationalization for feasibility in a German epidemiologic cohort study. The questionnaire modules reflected necessary prerequisites to measure sex/gender as a multidimensional, non-binary, intersectional construct with its entanglement of biological and social dimensions. The operationalization included an individual sex/gender self-concept, items assessing sex/gendered (power) relations and discrimination as well as lifestyle and psychosocial factors that help to specify intersectional sex/gender groups. However, in some cases, appropriate instruments still need to be developed. Nevertheless, our operationalization represents a new tool for collecting comprehensive quantitative data on sex/gender. Along with the given implications for statistical analysis, we paved the way for an adequate consideration of sex/gender in future environmental health research.

## Data availability statement

The datasets presented in this article are not readily available because an official request for project agreement is needed to receive the data. Requests to access the datasets should be directed to UK, ute.kraus@helmholtz-munich.de.

## Ethics statement

The studies involving human participants were reviewed and approved by Ethics Committees of the Bavarian Chamber of Physicians. The patients/participants provided their written informed consent to participate in this study.

## Author contributions

UK and AS collected the data. UK, LD, and GB cleaned the data and organized the database. UK and SF performed the statistical analysis. UK and KJ wrote the first draft of the manuscript. All authors contributed to conception and design of the study. All authors contributed to manuscript revision, read, and approved the submitted version.

## References

[1] Fausto-Sterling A. Sex/gender: Biology in a Social World. NY: Routledge (2012). 10.4324/9780203127971

[2] Day S Mason R Lagosky S Rochon PA. Integrating and evaluating sex and gender in health research. Health Res Policy Syst. (2016) 14:1–5. 10.1186/s12961-016-0147-7 27724961 PMC5057373

[3] Hammarström A Johansson K Annandale E Ahlgren C Aléx L Christianson M. Central gender theoretical concepts in health research: the state of the art. J Epidemiol Community Health. (2014) 68:185–90. 10.1136/jech-2013-202572 24265394

[4] Heidari S Babor TF De Castro P Tort S Curno M. Sex and gender equity in research: rationale for the SAGER guidelines and recommended use. Res Integr Peer Rev. (2016) 1:1–9. 10.1186/s41073-016-0007-6 29451543 PMC5793986

[5] Johnson JL Greaves L Repta R. Better science with sex and gender: facilitating the use of a sex and gender-based analysis in health research. Int J Equity Health. (2009) 8:9274. 10.1186/1475-9276-8-14 19419579 PMC2689237

[6] Krieger N. Genders, sexes, and health: what are the connections—and why does it matter? Int J Epidemiol. (2003) 32:652–7. 10.1093/ije/dyg156 12913047

[7] Oliffe JL Greaves L. Designing and Conducting Gender, Sex, and Health Research. London: Sage Publications, Inc. (2012).

[8] Bolte G. Geschlecht, Umwelt und Gesundheit. In:Kolip P Hurrelmann K, editors. Handbuch Geschlecht und Gesundheit Männer und Frauen im Vergleich. 2. Bern: Hogrefe AG (2016). p. 58–70.

[9] Clougherty JE A. growing role for gender analysis in air pollution epidemiology. Environ Health Perspect. (2010) 118:167–76. 10.1289/ehp.0900994 20123621 PMC2831913

[10] Ritz SA Greaves L. Transcending the male–female binary in biomedical research: constellations, heterogeneity, and mechanism when considering sex and gender. Int J Environ Res Public Health. (2022) 19:4083. 10.3390/ijerph19074083 35409764 PMC8998047

[11] Springer KW Stellman JM Jordan-Young RM. Beyond a catalogue of differences: a theoretical frame and good practice guidelines for researching sex/gender in human health. Soc Sci Med. (2012) 74:1817–24. 10.1016/j.socscimed.2011.05.033 21724313

[12] Horstmann S Schmechel C Palm K Oertelt-Prigione S Bolte G. The operationalization of sex and gender in quantitative health–related research: a scoping review. Int J Environ Res Public Health. (2022) 19:7493. 10.3390/ijerph19127493 35742742 PMC9224188

[13] Alexander AC Bolzendahl C Wängnerud L. Beyond the binary: new approaches to measuring gender in political science research. Eur J Polit Gender. (2021) 4:7–9. 10.1332/251510820X16067519822351

[14] Saperstein A Westbrook L. Categorical and gradational: Alternative survey measures of sex and gender. Eur J Polit Gender. (2021) 4:11–30. 10.1332/251510820X15995647280686

[15] FCSM. Federal Interagency Working Group on Improving Measurement of Sexual Orientation and Gender Identity in Federal Surveys. Evaluations of Sexual Orientation and Gender Identity Survey Measures: What Have We Learned? Federal Committee on Statistical Methodology (2016).

[16] The GenIUSS Group. Best Practices for Asking Questions to Identify Transgender and Other Gender Minority Respondents on Population-Based Surveys. Los Angeles, CA: The Williams Institute (2014).

[17] Bauer GR Braimoh J Scheim AI Dharma C. Transgender-inclusive measures of sex/gender for population surveys: Mixed-methods evaluation and recommendations. PLoS ONE. (2017) 12:e0178043. 10.1371/journal.pone.0178043 28542498 PMC5444783

[18] Hart CG Saperstein A Magliozzi D Westbrook L. Gender and health: Beyond binary categorical measurement. J Health Soc Behav. (2019) 60:101–18. 10.1177/0022146519825749 30698460

[19] Nielsen MW Stefanick ML Peragine D Neilands TB Ioannidis J Pilote L. Gender-related variables for health research. Biol Sex Differ. (2021) 12:23. 10.1186/s13293-021-00366-3 33618769 PMC7898259

[20] Pelletier R Ditto B Pilote L A. composite measure of gender and its association with risk factors in patients with premature acute coronary syndrome. Psychosom Med. (2015) 77:517–26. 10.1097/PSY.0000000000000186 25984818

[21] Nauman AT Behlouli H Alexander N Kendel F Drewelies J Mantantzis K. Gender score development in the Berlin Aging Study II: a retrospective approach. Biol Sex Differ. (2021) 12:15. 10.1186/s13293-020-00351-2 33461607 PMC7814714

[22] Beischel WJ Schudson ZC van Anders SM. “This Is Mind Expanding”: Reactions to an Online Survey Using Sexual Configurations Theory. Psychol Sexual Orient Gender Diver. (2021) 8:14–24. 10.1037/sgd0000450

[23] Beischel WJ Schudson ZC van Anders SM. Visualizing gender/sex diversity via sexual configurations theory. Psychol Sexual Orient Gender Diver. (2021) 8:1–13. 10.1037/sgd0000449

[24] Bauer GR Churchill SM Mahendran M Walwyn C Lizotte D Villa-Rueda AA. Intersectionality in quantitative research: a systematic review of its emergence and applications of theory and methods. SSM-Populat Health. (2021) 14:100798. 10.1016/j.ssmph.2021.100798 33997247 PMC8095182

[25] Mena E Bolte G Group AS. CART-analysis embedded in social theory: a case study comparing quantitative data analysis strategies for intersectionality-based public health monitoring within and beyond the binaries. SSM-Populat Health. (2021) 13:100722. 10.1016/j.ssmph.2020.100722 33385059 PMC7772559

[26] Bolte G Jacke K Groth K Kraus U Dandolo L Fiedel L. Integrating sex/gender into environmental health research: development of a conceptual framework. Int J Environ Res Public Health. (2021) 18:12118. 10.3390/ijerph182212118 34831873 PMC8621533

[27] Krieger N. Embodiment: a conceptual glossary for epidemiology. J Epidemiol Community Health. (2005) 59:350–5. 10.1136/jech.2004.024562 15831681 PMC1733093

[28] Schröter-Kermani C Gies A Kolossa-Gehring M. Die Umweltprobenbank des Bundes. Bundesgesundheitsblatt-Gesundheitsforschung-Gesundheitsschutz. (2016) 59:368–72. 10.1007/s00103-015-2298-z 26753867

[29] Holle R Happich M Löwel H Wichmann H-E Group MKS. KORA-a research platform for population based health research. Das Gesundheitswesen. (2005) 67:19–25. 10.1055/s-2005-858235 16032513

[30] Delbecq AL Van de Ven AH A. group process model for problem identification and program planning. J Appl Behav Sci. (1971) 7:466–92. 10.1177/002188637100700404

[31] Denton M Prus S Walters V. Gender differences in health: a Canadian study of the psychosocial, structural and behavioural determinants of health. Soc Sci Med. (2004) 58:2585–600. 10.1016/j.socscimed.2003.09.008 15081207

[32] Reid AE Cialdini RB Aiken LS. Social Norms and Health Behavior. In:Steptoe A, editor. Handbook of behavioral medicine: Methods and applications. New York, NY: Springer (2011). p. 263–74. 10.1007/978-0-387-09488-5_19

[33] Beigang S Fetz K Kalkum D Otto M. Diskriminierungserfahrungen in Deutschland: Ergebnisse einer Repräsentativ-und einer Betroffenenbefragung. Antidiskriminierungsstelle des Bundes. (2017). Report No.: 3848746751.

[34] Tate CC Ledbetter JN Youssef CP A. two-question method for assessing gender categories in the social and medical sciences. J Sex Res. (2013) 50:767–76. 10.1080/00224499.2012.690110 22989000

[35] Kachel S Steffens MC Niedlich C. Traditional masculinity and femininity: Validation of a new scale assessing gender roles. Front Psychol. (2016) 7:956. 10.3389/fpsyg.2016.00956 27458394 PMC4932111

[36] ISSP. International social survey programme: Family and changing gender roles IV. (2012). Contract No.: July 15th, 2022.

[37] Scholz E Jutz R. ISSP 2012 Germany: Family and Gender Roles IV; GESIS Report on the German Study (GESIS-Technical Reports, 2014/11). Köln: GESIS - Leibniz-Institut für Sozialwissenschaften (2014). Report No.: 1868-9051.

[38] Gordon AR Krieger N Okechukwu CA Haneuse S Samnaliev M Charlton BM. Decrements in health-related quality of life associated with gender nonconformity among US adolescents and young adults. Qual Life Res. (2017) 26:2129–38. 10.1007/s11136-017-1545-1 28315179 PMC5511094

[39] Wylie SA Corliss HL Boulanger V Prokop LA Austin SB. Socially assigned gender nonconformity: A brief measure for use in surveillance and investigation of health disparities. Sex Roles. (2010) 63:264–76. 10.1007/s11199-010-9798-y 24077680 PMC3783339

[40] EWCS. European Working Conditions Surveys 2015. European Foundation for the Improvement of Living and Working Conditions. (2015). Contract No.: July 14th, 2022.

[41] SVR. Viele Götter, ein Staat: Religiöse Vielfalt und Teilhabe im Einwanderungsland. Jahresgutachten 2016 mit Integrationsbarometer. Berlin, Germany: Sachverständigenrat deutscher Stiftungen für Integration und Migration (SVR) GmbH (2016).

[42] SOEP. Sozio-ökonomisches Panel. (2017). Contract No.: July 18th, 2022.

[43] EQLS. European Quality of Life Survey. European Foundation for the Improvement of Living and Working Conditions. (2016). Contract No.: August 8th, 2022.

[44] SHARE. Survey of Health, Ageing and Retirement in Europe. CAPI main questionnaire.; (n.d.) Contract No.: August 8th, 2022.

[45] Klemmer CL Rusow J Goldbach J Kattari SK Rice E. Socially assigned gender nonconformity and school violence experience among transgender and cisgender adolescents. J Interpers Violence. (2021) 36:NP8567–NP89. 10.1177/0886260519844781 31023178

[46] Lagos D Compton DL. Evaluating the use of a two-step gender identity measure in the 2018 General Social Survey. Demography. (2021) 58:763–72. 10.1215/00703370-8976151 33834217 PMC9084897

[47] Baumann A-L Egenberger V Supik L. Erhebung von Antidiskriminierungsdaten in repräsentativen Wiederholungsbefragungen. Bestandsaufnahme und Entwicklungsmöglichkeiten. Berlin, Germany: Antidiskriminierungsstelle des Bundes. (2018).

[48] Beigang S Fetz K Foroutan N Kalkum D Otto M. Diskriminierungserfahrungen in Deutschland: Erste Ergebnisse einer repräsentativen Erhebung und einer Betroffenenbefragung. Berlin, Germany: Antidiskriminierungsstelle des Bundes. (2016).

[49] The GenIUSS Group. Gender-related Measures Overview. Los Angeles, CA: The Williams Institute (2013).

[50] Gates GJ. How many people are lesbian, gay, bisexual and transgender? (2011). Contract No.: July 15th, 2022.

[51] Meerwijk EL Sevelius JM. Transgender population size in the United States: a meta-regression of population-based probability samples. Am J Public Health. (2017) 107:e1–8. 10.2105/AJPH.2016.303578 28075632 PMC5227946

[52] Nolan IT Kuhner CJ Dy GW. Demographic and temporal trends in transgender identities and gender confirming surgery. Transl Androl Urol. (2019) 8:184–90. 10.21037/tau.2019.04.09 31380225 PMC6626314

[53] Bem SL. The measurement of psychological androgyny. J Consult Clin Psychol. (1974) 42:155–62. 10.1037/h0036215 4823550

[54] Smiler A Epstein M. Issues in the measurement of gender. Handbook Gender Res Psychol. (2010) 1:133–58. 10.1007/978-1-4419-1465-1_7

[55] Wängnerud L Solevid M Djerf-Pierre M. Moving beyond categorical gender in studies of risk aversion and anxiety. Politics Gender. (2019) 15:826–50. 10.1017/S1743923X18000648

[56] Berkman LF Kawachi I Glymour MM. Social epidemiology. Oxford: Oxford University Press (2014). 10.1093/med/9780195377903.001.0001

[57] Johnson SC Cavallaro FL Leon DA A. systematic review of allostatic load in relation to socioeconomic position: poor fidelity and major inconsistencies in biomarkers employed. Soc Sci Med. (2017) 192:66–73. 10.1016/j.socscimed.2017.09.025 28963986

[58] Gordon AR Meyer IH. Gender nonconformity as a target of prejudice, discrimination, and violence against LGB individuals. J LGBT Health Res. (2007) 3:55–71. 10.1080/15574090802093562 19042905 PMC10790306

[59] Miller LR Grollman EA. The social costs of gender nonconformity for transgender adults: Implications for discrimination and health. Sociol Forum. (2015) 30:809–31. 10.1111/socf.12193 27708501 PMC5044929

[60] Lewis TT Cogburn CD Williams DR. Self-reported experiences of discrimination and health: scientific advances, ongoing controversies, and emerging issues. Annu Rev Clin Psychol. (2015) 11:407–40. 10.1146/annurev-clinpsy-032814-112728 25581238 PMC5555118

[61] Aikins JK Supik L. Gleichstellungsdaten: Differenzierte Erfassung als Grundlage für menschenrechtsbasierte Antidiskriminierungspolitik. In:Foroutan N Karakayali J Spielhaus R, editors. Postmigrantische Perspektiven. Frankfurt am Main/New York: Campus Verlag (2018). p. 97–112.

[62] El-Mafaalani A Waleciak J Weitzel G. Tatsächliche, messbare und subjektiv wahrgenommene Diskriminierung. Handbuch Diskriminierung: Springer (2017). p. 173–89. 10.1007/978-3-658-10976-9_10

[63] Bundesregierung. Zweiter Gleichstellungsbericht der Bundesregierung. Berlin, Germany: BT-Drucksache (2017).

[64] Schulz R Sherwood PR. Physical and mental health effects of family caregiving. J Soc Work Educ. (2008) 44:105–13. 10.5175/JSWE.2008.773247702 18797217 PMC2791523

[65] Xue B McMunn A. Gender differences in unpaid care work and psychological distress in the UK Covid-19 lockdown. PLoS ONE. (2021) 16:e0247959. 10.1371/journal.pone.0247959 33662014 PMC7932161

[66] Ehrenreich B Hochschild AR. Global Woman: Nannies, Maids, and Sex Workers in the New Economy. New York: Metropolitan Books. (2003).

[67] Lutz H. Intime Fremde – Migrantinnen als Haushaltsarbeiterinnen in Westeuropa. L'homme: Zeitschrift für feministische Geschichtswissenschaft. (2007) 18:61–78. 10.7767/lhomme.2007.18.1.61

[68] Hoffmann W Latza U Baumeister SE Brünger M Buttmann-Schweiger N Hardt J. Guidelines and recommendations for ensuring Good Epidemiological Practice (GEP): a guideline developed by the German Society for Epidemiology. Eur J Epidemiol. (2019) 34:301–17. 10.1007/s10654-019-00500-x 30830562 PMC6447506

[69] Rioux C Paré A London-Nadeau K Juster RP Weedon S Levasseur-Puhach S. Sex and gender terminology: a glossary for gender-inclusive epidemiology. J Epidemiol Community Health. (2022) 76:764–8. 10.1136/jech-2022-219171 35725304

[70] Pöge K Dennert G Koppe U Güldenring A Matthigack EB Rommel A. The health of lesbian, gay, bisexual, transgender and intersex people. J Health Monitoring. (2020) 5:2–27. 10.25646/6449 35146279 PMC8734091

[71] Matthiesen S Dekker A Brunner F Klein V Martyniuk U Schmidt D. Sexuelles Verhalten, Einstellungen und sexuelle Gesundheit in Deutschland: Erste Ergebnisse einer Pilotstudie zur Erwachsenensexualität. UKE Hamburg, BZgA, Kantar Emnid. (2017).

[72] Cerwenka S Brunner F. Sexuelle Identität, sexuelle Attraktion und sexuelles Verhalten–Dimensionen sexueller Orientierungen in der Survey-Forschung. Zeitschrift für Sexualforschung. (2018) 31:277–94. 10.1055/a-0664-4764

[73] Israel BA Eng E Schulz AJ Parker EA. Introduction to methods in community-based participatory research for health. Methods Commun Based Participat Res Health. (2005) 3:26.35321219

[74] Bauer GR. Incorporating intersectionality theory into population health research methodology: challenges and the potential to advance health equity. Soc Sci Med. (2014) 110:10–7. 10.1016/j.socscimed.2014.03.022 24704889

[75] Merz S Jaehn P Mena E Pöge K Strasser S Saß A-C. Intersectionality and eco-social theory: a review of potentials for public health knowledge and social justice. Crit Public Health. (2021) 33:125–34. 10.1080/09581596.2021.1951668

[76] Mena E Bolte G Group AS. Intersectionality-based quantitative health research and sex/gender sensitivity: a scoping review. Int J Equity Health. (2019) 18:199. 10.1186/s12939-019-1098-8 31864366 PMC6925460

[77] Bolte G David M Debiak M Fiedel L Hornberg C Kolossa-Gehring M. Integration von Geschlecht in die Forschung zu umweltbezogener Gesundheit: Ergebnisse des interdisziplinären Forschungsnetzwerks Geschlecht–Umwelt–Gesundheit (GeUmGe-NET). Bundesgesundheitsblatt-Gesundheitsforschung-Gesundheitsschutz. (2018) 61:737–46. 10.1007/s00103-018-2745-8 29789893

[78] Dandolo L Hartig C Telkmann K Horstmann S Schwettmann L Selsam P. Decision tree analyses to explore the relevance of multiple sex/gender dimensions for the exposure to green spaces: results from the KORA INGER study. Int J Environ Res Public Health. (2022) 19:7476. 10.3390/ijerph19127476 35742725 PMC9224469

