# Supplementary material for: Operationalization of a multidimensional sex/gender concept for quantitative environmental health research and implementation in the KORA study: Results of the collaborative research project INGER
Source: Front Public Health. 2023 Apr 17;11:1128918. doi: 10.3389/fpubh.2023.1128918 (PMC10152671; doi:10.3389/fpubh.2023.1128918)
Supplement: Supplementary file 1 [file Data_Sheet_1.pdf]

Supplementary Material

Operationalization of a multidimensional sex/gender concept for quantitative environmental health research and implementation in the KORA study: Results of the collaborative research project INGER

Ute Kraus*, Katharina Jacke, Lisa Dandolo, Malgorzata Debiak, Sophie Fichter, Katrin Groth, Marike Kolossa-Gehring, Christina Hartig, Sophie Horstmann, Alexandra Schneider, Kerstin Palm, Gabriele Bolte

*** Correspondence:** Ute Kraus: ute.kraus@helmholtz-munich.de

Contents

S1. Intersectionality-related social categories and its frequency distribution in the KORA cohort
S2. Lifestyle and psychological factors and its frequency distribution in the KORA cohort
S3. Overview of eligible KORA participants
S4. Unsolicited comments on sex/gender questions
S5. Descriptive results of internalized sex/gender roles and externalized sex/gender expressions
S6. Descriptive results on (non)conformity
S7. Descriptive results on attitudes towards gender roles (N=3,472)
S8. Descriptive results on experiences of discrimination (N=3,472)
S9. Descriptive results of care and household activities (N=3,472)
S10. Missing rates in sex/gender variables

S1. Intersectionality-related social categories and its frequency distribution in the KORA cohort

Item	Coding	N	% (if not other specified)
**Family situation**[1]	Living alone	488	18.6
	Living without partner, living together with other persons	60	2.3
	Living with partner without other persons	1,619	61.7
	Living with partner with other persons	455	17.3
	Missing	2	0.1
**Education**			
School education	Secondary school, basic certificate	1,736	46.4
	Secondary school, middle maturity	1,085	29.0
	A-levels	920	24.6
	Missing	1	0.0
Highest vocational qualification	No vocational qualification	272	7.3
	Vocational school / apprenticeship	2,064	55.2
	Technical school / master school	725	19.4
	Engineering school/polytechnic school	32	0.9
	University of Applied Sciences /University	648	17.3
	Missing	1	0.0
**Occupation**[1]	Unskilled worker	52	2.0
	Civil servant simple activity / semi-skilled worker	115	4.4
	Civil servant simple service / skilled worker	396	15.1
	Civil servant middle service / skilled worker or foreman	133	5.1
	Master craftsman / foreman	43	1.6
	Civil servants higher service / employee with qualified activity	1,040	39.6
	Self-employed, max. 1 employee	162	6.2
	Civil servant higher service / employee with highly qualified activity / self-employed academic / self-employed with max. 9 employees	541	20.6
	Employees with extensive managerial responsibilities / self-employed with at least 10 employees	79	3.0
	Missing	63	2.4
**Employment**			
Retirement status	yes	1,785	47.7
	No	1,498	40.0
	Unclear, but participant is of retirement age (>=66 years)	202	5.4
	Unclear, but participant is not of retirement age (<66 years)	257	6.9
	Missing	0	0
Are you employed?	No	1,970	52.6
	0 - 9 h/week	103	2.8

If so: How many hours do you work on average per week (actual working hours)?	10 - 19 h/ week	151	4.0
	20 - 29 h/ week	252	6.7
	30 - 39 h/ week	422	11.3
	40 - 49 h/ week	544	14.5
	>= 50 h/ week	209	5.6
	Employed, no hours specified	20	0.5
	Missing	71	1.9
**Income**			
How do you assess your financial situation?	Very good	394	10.5
	Good	2,303	61.5
	Moderate	937	25.0
	Bad	66	1.8
	Missing	42	1.1
Net household income - mean values per income group [€]	333	7	0.3
	625	11	0.4
	875	46	1.8
	1250	174	6.6
	1750	257	9.8
	2250	348	13.3
	2750	333	12.7
	3250	323	12.3
	3750	212	8.1
	4250	205	7.8
	4750	115	4.4
	5250	125	4.8
	5750	75	2.9
	8000	198	7.5
	Missing	195	7.4
Equivalent income[1]	Continuous [€]	Mean<br>1,902	SD<br>923
	Missing	195	7.4
**Social position[1]**			
Helmert index (quintiles)	1 – 10 points (lower status)	531	20.2
	>10 - 13 points	593	22.6
	>13 - 15 points	447	17.0
	>15 - 19 points	538	20.5
	> 19 points (upper status)	506	19.3
	Missing	9	0.3
**Ethnicity**			
Born in Germany[2]	Yes	1,516	87.9
	No	208	12.1
	Missing	1	0.1
Origin of grandparents[3]	German grandparent	1,356	81.4

	Non-German or 1 unknown grandparent	83	5.0
	More than 1 unknown grandparent	172	10.3
	Missing	54	3.2
**Disability**[1]			
Do you have a recognized disability?	Yes	511	19.5
	No	2,112	80.5
	Missing	2	0.0
If yes, what degree of disability do you have?	Continuous (%)	Mean<br>48.7	SD<br>19.0
	Missing	1	0.2

[1] data collected only in KORA FIT, N=2,624
[2] data collected only in KORA S4, N=1,725
[3] data collected only in KORA F4, N=1,665
SD, standard deviation

S2. Lifestyle and psychosocial factors and its frequency distribution in the KORA cohort

Item	Coding	N (if not other specified)	%
**Smoking status**[1]	Regular smoker	303	11.5
	Irregular smoker	43	1.6
	Ex-smoker	1,085	41.3
	Never smoker	1,191	45.4
	Missing	2	0.1
**Alcohol consumption**[1]			
How often do you consume alcoholic beverages? Please think about the last 12 months.	Daily or almost daily	529	20.2
	Several times a week	640	24.4
	About once a week	469	17.9
	Less frequently than once a week	382	14.6
	Never or extremely rarely	602	22.9
	Missing	2	0.1
**Physical activity**[1]	Regularly more than 2 hours a week	1,021	38.9
	Regularly approx. 1 hour a week	889	33.9
	Irregularly approx. 1 hour per week	322	12.3
	Almost no or no physical activity	392	14.9
	Missing	0	0.0
**Stress**[1]			
PSS - Perceived stress scale (Cohen et al. 1983)	Continuous (Range 0-40) with a higher score corresponding to a higher stress level	Mean 14.3	SD 5.5
	Missing	126	2.0
**Self-efficacy**[1]			
GSE – General self-efficacy Short Scale-3 (ASKU, "Allgemeine Selbstwirksamkeit Kurzskala", (Beierlein et al. 2012)	Continuous (Range 1-5) with a higher score corresponding to a higher self-efficacy	Mean 4.0	SD 0.6
	Missing	76	1.2

[1] data collected only in KORA FIT, N=2,624

S3. Overview of eligible KORA participants

	S1	S2	S3	S4
	1984/85	1989/90	1994/95	1999/2000
**Born in**				
**1915 - 1919**		✓		
**1920 - 1924**	✓	✓	✓	
**1925 - 1929**	✓	✓	✓	✓
**1930 - 1934**	✓	✓	✓	✓
**1935 - 1939**	✓	✓	✓	✓
**1940 - 1944**	✓	✓	✓	✓
**1945 - 1949**	✓	✓	✓	✓
**1950 - 1954**	✓	✓	✓	✓
**1955 - 1959**	✓	✓	✓	✓
**1960 - 1964**		✓	✓	✓
**1965 - 1969**			✓	✓
**1970 - 1975**				✓

Dark blue: KORA FIT participants, light blue: younger participants of S3 and all other participants of S4

S4. Unsolicited comments on sex/gender questions

Internalized sex/gender roles and externalized sex/gender expressions

Questions:
a) I mostly perceive myself as....
b) Ideally I would like to be…"
c) How would other people generally describe you based on your appearance, clothing style, and other visual characteristics?
d) How would other people generally describe you based on your behaviors?"

Reference	Unsolicited Comments
In general:	○ What are these questions about?<br>○ Definition?<br>○ Bullshit<br>○ I feel like a man!<br>○ How do they define "feminine"? I cannot do anything with question 8!!!<br>○ Term I do not know<br>○ What is masculine, what is feminine?<br>○ I never ask myself that!?<br>○ Question not understood
Regarding a)	○ ??
Regarding b)	○ Gender nonsense<br>○ Would be? I am!<br>○ ??<br>○ How I like it<br>○
Regarding c)	○ Response denied<br>○ ??<br>○ Very sporty<br>○ Not masculine stupid question<br>○ Sporty
Regarding d)	○ Do not understand this question<br>○ Hopefully as an empathetic, respectful, loving person!!!<br>○ Very sporty<br>○ I don't know

Fluidity of sex/Gender

Question: “Has your assessment of what is feminine or masculine changed in recent years?”

- Nonsense!
- Stupid questions. Can't do anything with them.
- The role model of man and woman has changed, for me the human being counts, no matter if man or woman
- There is more difference in the same sex than between the sexes! For me the qualities count, not male, female
- I don't answer questions from 8 to 9 because I don't understand them

Current sex/gender identity

Question: “What is your current sex/gender identity?”

- No information
- Normal
- Joke come out
- Current? → such nonsense, gender is not a consumer good!
- Sporty

Sex assigned at birth

“What sex were you assigned at birth?”

- Bullshit!

Internalized sex/gender roles

Nine statements on attitudes on gender:

Statement	Comments
“Both the men and women should both contribute to the household income.”	o But cannot himself (disabled)<br>o Depending on consumption
“The man's job is to earn money; a woman's job is to look after the home and family.”	o Both
“A working mother can establish just as warm and secure a relationship with her children as a mother who does not work.”	o Overstrained?<br>o Nonsense!<br>o Work half day
“A pre-school child is likely to suffer if his or her mother works.”	o Both parents are fully employed<br>o One parent?

	○ Depends on how you handle it<br>○ Depends on the scope of work
"All in all, family life suffers when the woman is working."	○ Both parents are employed.<br>○ Wife ? Parent ?<br>○ Part-time
"Being a housewife is just as fulfilling as working for pay."	○ Housewife and mother<br>○ ?<br>○ Can / be<br>○ Can<br>○ Should be paid<br>○ When the appropriate appreciation is made
"Being a househusband is just as fulfilling as working for pay."	○ Not always<br>○ Househusband and father<br>○ ?<br>○ Can / be<br>○ Can<br>○ Should be paid<br>○ Only a man can judge<br>○ When the appropriate appreciation is made
"One parent can bring up a child as well as two parents together."	○ Inevitably
"A same sex couple can bring up a child as well as a male-female couple."	○ Immoral, no further comment<br>○ What are these questions for?<br>○ Children are teased at some point and suffer for it!<br>○ No comment

S5. Descriptive results of internalized sex/gender roles and externalized sex/gender expressions (N=3,472)

"The following statements relate to how feminine or masculine you perceive yourself to be. Please describe yourself by selecting the answer that best fits you."		very masculine	mainly masculine	little masculine	as feminine as masculine	a little feminine	mainly feminine	very feminine	missing
"I mostly perceive myself as..."	N	403	866	303	426	173	958	436	177
	%	10.8	23.1	8.1	11.4	4.6	25.6	11.7	4.7
"Ideally I would like to be…"	N	482	844	196	384	151	878	550	257
	%	12.9	22.6	5.2	10.3	4.0	23.5	14.7	6.9
"How would other people generally describe you based on your appearance, clothing style, and other visual characteristics?"	N	413	884	256	326	253	1,012	388	210
	%	11.0	23.6	6.8	8.7	6.8	27.0	10.4	5.6
"How would other people generally describe you based on your behaviors?"	N	384	876	301	455	232	953	338	203
	%	10.3	23.4	8.0	12.2	6.2	25.5	9.0	5.4

S6. Descriptive results on socially assigned sex/gender (non)conformity

		Assumed appraisal of others			
		Conforming	Non-Conforming	Missing	In total
Self-rated appraisal	Conforming	3.373 (90.1)	54 (1.4)	43 (1.1)	3.470 (92.7)
	Non-conforming	32 (0.9)	39 (1.0)	4 (0.1)	75 (2.0)
	Missing	8 (0.2)	0 (0.0)	189 (5.1)	197 (5.3)
	In total	3.413 (91.2)	93 (2.5)	236 (6.3)	3.742 (100.0)

S7. Descriptive results on attitudes towards gender roles (N=3,472)

Attitudes towards gender roles:		Fully agree	Rather agree	Partly/ partly	Rather disagree	Fully disagree	Missing
"Both the men and women should contribute to the household income."	N	1,766	990	801	95	33	57
	%	47.2	26.5	21.4	2.5	0.9	1.5
"The man's job is to earn money; a woman's job is to look after the home and family."	N	106	293	898	1,010	1,380	55
	%	2.8	7.8	24.0	27.0	36.9	1.5
"A working mother can establish just as warm and secure a relationship with her children as a mother who does not work."	N	1,840	972	610	213	52	55
	%	49.2	26.0	16.3	5.7	1.4	1.5
"A pre-school child is likely to suffer if his or her mother works."	N	424	1.002	1.112	730	414	60
	%	11.3	26.8	29.7	19.5	11.1	1.6
"All in all, family life suffers when the woman is working."	N	150	568	1,377	993	596	58
	%	4.0	15.2	36.8	26.5	15.9	1.5
"Being a housewife is just as fulfilling as working for pay."	N	520	622	1,089	1,013	430	68
	%	13.9	16.6	29.1	27.1	11.5	1.8
"Being a househusband is just as fulfilling as working for pay."	N	318	468	1,045	1,208	579	124
	%	8.5	12.5	27.9	32.3	15.5	3.3
"One parent can bring up a child as well as two parents together."	N	377	633	1,181	1,139	363	49
	%	10.1	16.9	31.6	30.4	9.7	1.3
"A same sex couple can bring up a child as well as a male-female couple."	N	667	838	898	708	567	64
	%	17.8	22.4	24.0	18.9	15.2	1.7

S8. Descriptive results on experiences of discrimination (N=3,472)

"I have the feeling…"		Fully applies	Rather applies	Partly/ partly	Rather does not apply	Does not apply at all	Missing
"…that life offers me many opportunities."	N	1,386	1,040	1,046	193	32	45
	%	37.0	27.8	28.0	5.2	0.9	1.2
"…to be accepted as I am."	N	1,669	1,262	676	91	9	35
	%	44.6	33.7	18.1	2.4	0.2	0.9
"…being heavily involved due to family responsibilities."	N	1,058	1,021	952	509	159	46
	%	28.3	27.3	25.4	13.6	4.2	1.1
"…to be disadvantaged by my position in society"	N	40	202	354	1,379	1,717	50
	%	1.1	5.4	9.5	36.9	45.9	1.3
"...to be disadvantaged because of my age."	N	49	251	431	1,343	1,630	38
	%	1.3	6.7	11.5	35.9	43.6	1.0
"...to be disadvantaged because of my height."	N	23	71	143	922	2,544	39
	%	0.6	1.9	3.8	24.6	68.0	1.0
"...to be disadvantaged because of my weight."	N	29	75	187	888	2,521	42
	%	0.8	2.0	5.0	23.7	67.4	1.1
"...to be disadvantaged because of my physical impairment."	N	46	95	228	644	2,681	48
	%	1.2	2.5	6.1	17.2	71.6	1.3
"...to be disadvantaged because of my ethnic/cultural affiliation."	N	14	25	56	408	3,185	54
	%	0.4	0.7	1.5	10.9	85.1	1.4
"...to be disadvantaged because of my sex/gender."	N	17	42	128	495	3,018	42
	%	0.5	1.1	3.4	13.2	80.7	1.1
"...to be disadvantaged because of my sexual orientation."	N	13	6	25	251	3,368	79
	%	0.3	0.2	0.7	6.7	90.0	2.1
		Yes	No	Missing			
"…to be disadvantaged because of other reasons, namely:"	N	104	3.638	--			
	%	2.8%	97.2%	--			
"Have you ever been asked in Germany whether you or your parents were born abroad?"	N	417	3,280	45			
	%	11.1	87.7	1.2			

		Physical appearance	Clothes	Religious signs	Name	Language accent	Nutrition	Other
"If so, what characteristics do you think they asked you about?" (N=415)	N	94	6	9	160	161	12	48
	%	22.4	1.34	2.2	38.4	38.6	2.9	11.5

S9. Descriptive results of care and household activities (N=3,472)

"Who is currently taking primary responsibility for the following tasks? ("Other people" also includes your partner)"		Only me	Mainly me	I together with other persons	Mainly other persons	Only other persons	Does not apply	Missing
Care and/or upbringing of your children/grandchildren, driving services for your children/grandchildren	N	155	329	1,173	393	71	1,572	49
	%	4.1	8.8	31.3	10.5	1.9	42.0	1.3
Care for disabled, chronically ill or in need of care family members, neighbors or friends	N	166	275	571	247	77	2,342	65
	%	4.4	7.3	15.3	6.6	2.1	62.6	1.7
Earning a living (including pension)	N	811	576	1,793	236	54	0	272
	%	21.7	15.4	47.9	6.3	1.4	0.0	7.3
Cooking	N	1,049	756	877	650	348	0	62
	%	28.0	20.2	23.4	17.4	9.3	0.0	1.7
Housework	N	896	877	1,200	629	98	0	42
	%	23.9	23.4	32.1	16.8	2.6	0.0	1.1
Errands (shopping, procurement)	N	797	800	15,566	434	76	0	69
	%	21.3	21.4	41.8	11.6	2.0	0.0	1.8
Administrative tasks (insurance, tax return, etc.)	N	1,001	857	1,191	481	163	0	49
	%	26.8	22.9	31.8	12.9	4.4	0.0	1.3
Technical activities (e.g. computer, internet)	N	606	821	1,085	641	252	286	51
	%	16.2	21.9	29.0	17.1	6.7	7.6	1.4
Handicraft tasks in the household	N	704	936	906	850	303	0	43
	%	18.8	25.0	24.2	22.7	8.1	0.0	1.1
Gardening (during the gardening season)	N	495	686	1,449	423	111	535	43
	%	13.2	18.3	38.7	11.3	3.0	14.3	1.1

S10. Missing rates in sex/gender variables

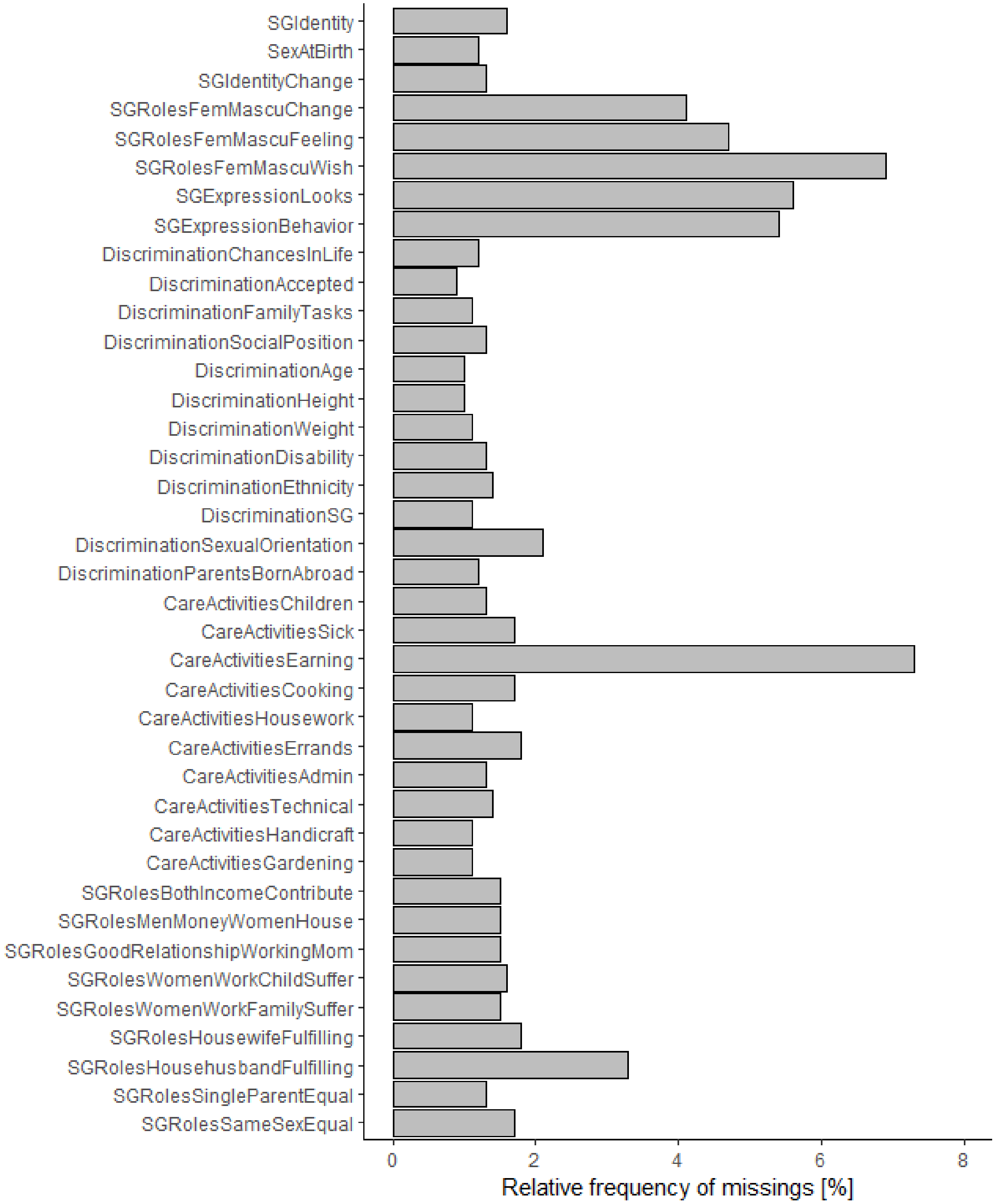

Explanation of variable names:
SGIdentity, What is your current sex/gender identity?
SexAtBirth, What sex was assigned to you at birth?
SGRolesFemMascuChange, Has your perception of “feminine” and “masculine” changed in recent years?
SGRolesFemMascuFeeling, I mostly see myself as …
SGRolesFemMascuWish, Ideally, I would like to be ...
SGExpressionLooks, In general, how would other people describe you based on your appearance, clothing style and other visual characteristics?
SGExpressionBehavior, In general, how would other people describe you based on your behavior?
DiscriminationChancesInLife, I have the feeling that life offers me many opportunities.
DiscriminationAccepted, I have the feeling to be accepted as I am.
DiscriminationFamilyTasks, I have the feeling being heavily involved due to family responsibilities.
DiscriminationSocialPosition, I have the feeling to be disadvantaged because of my position in society.
DiscriminationAge, I have the feeling to be disadvantaged because of my age.
DiscriminationHeight, I have the feeling to be disadvantaged because of my height.
DiscriminationWeight, I have the feeling to be disadvantaged because of my weight.
DiscriminationDisability, I have the feeling to be disadvantaged because of my physical impairment.
DiscriminationEthnicity, I have the feeling to be disadvantaged because of my ethnic/cultural affiliation.
DiscriminationSG, I have the feeling to be disadvantaged because of my sex/gender.
DiscriminationSexualOrientation, I have the feeling to be disadvantaged because of my sexual orientation.
DiscriminationParentsBornAbroad, Have you ever been asked in Germany whether you or your parents were born abroad?
CareActivitiesChildren, Care and/or upbringing of your children/grandchildren, driving services for your children/grandchildren
CareActivitiesSick, Care for disabled, chronically ill or in need of care family members, neighbours or friends
CareActivitiesEarning, Earning a living (including pension)
CareActivitiesCooking, Cooking
CareActivitiesHousework, Housework
CareActivitiesErrands, Errands (shopping, procurement)
CareActivitiesAdmin, Administrative tasks (insurance, tax return, etc.)
CareActivitiesTechnical, Technical activities (e.g. computer, internet)
CareActivitiesHandicraft, Handicraft tasks in the household
CareActivitiesGardening, Gardening (during the gardening season)
SGRolesBothIncomeContribute, The husband and wife should both contribute to the household income.
SGRolesMenMoneyWomenHouse, The man's role is to earn money, the woman's role to look after the household and family.
SGRolesGoodRelationshipWorkingMom, A working mother can have just as warm and trusting relationships with her children as a mother who does not work.
SGRolesWomenWorkChildSuffer, A child who is not yet in school is likely to suffer when their mother is working.
SGRolesWomenWorkFamilySuffer, All in all, family life suffers when the woman is working.
SGRolesHousewifeFulfilling, Being a housewife is just as fulfilling as working for money.
SGRolesHousehusbandFulfilling, Being a househusband is just as fulfilling as working for money.
SGRolesSingleParentEqual, A single parent can raise their child just as well as both parents together.

References

Beierlein C, Kovaleva A, Kemper C and Rammstedt B (2012). ASKU-Allgemeine Selbstwirksamkeit Kurzskala [Fragebogen]. Leibniz-Zentrum für Psychologische Information und Dokumentation. https://doi.org/10.23668/psycharchives.4527.

Cohen S, Kamarck T and Mermelstein R (1983). A global measure of perceived stress. J Health Soc Behav, 24(4): 385-396. https://doi.org/10.2307/2136404.
